# Bromocriptine treatment associated with recovery from peripartum cardiomyopathy in siblings: two case reports

**DOI:** 10.1186/1752-1947-4-80

**Published:** 2010-03-04

**Authors:** Gerd Peter Meyer, Saida Labidi, Edith Podewski, Karen Sliwa, Helmut Drexler, Denise Hilfiker-Kleiner

**Affiliations:** 1 Department of Cardiology and Angiology, MHH, Carl Neuberg Strasse 1, 30625 Hannover, Germany; 2 Soweto Cardiovascular Research Unit, Chris-Hani-Baragwanath Hospital, University of the Witwatersrand, Old Potch Road 100, 2013 Soweto, Johannesburg, South Africa

## Abstract

**Introduction:**

Peripartum cardiomyopathy is a rare form of cardiomyopathy, with heterogeneous presentation occurring in women between one-month antepartum and six months postpartum. It carries a poor prognosis and a high risk of mortality.

**Case presentation:**

We report the development of peripartum cardiomyopathy in two sisters, 27- and 35-year-old African women, one of whom presented with a large left ventricular thrombus. Subsequently, both patients were treated with bromocriptine, heparin and standard therapy for heart failure (angiotensin converting enzyme inhibitors, beta-blockers and diuretics). During follow-up, the left ventricular thrombus observed in one patient degraded. Neither patient experienced a thrombotic event, and both experienced continuous improvements in cardiac function and New York Heart Association stage.

**Conclusion:**

The development of peripartum cardiomyopathy in two sisters indicates that there may be a genetic basis for this type of cardiomyopathy, and that women with a positive family history for peripartum cardiomyopathy may have an increased risk of developing the disease. This is also the first report of a patient experiencing degradation of a large left ventricular thrombus under standard therapy for heart failure with bromocriptine. It suggests that the use of bromocriptine in association with adequate anti-coagulation and heart failure therapy may be beneficial and safe.

## Introduction

Peripartum cardiomyopathy is a distinct entity of dilated cardiomyopathy, and occurs in women between one month antepartum and six months postpartum. Specific risk factors for peripartum cardiomyopathy have not been defined and reports in the literature regarding positive family histories of peripartum cardiomyopathy are rare [1]. However, the higher incidence of peripartum cardiomyopathy in certain geographic areas, specifically Africa and Haiti, indicates a possible genetic factor [2].

At present, peripartum cardiomyopathy is treated according to the guidelines for dilated cardiomyopathy with angiotensin converting enzyme (ACE) inhibitors, beta-blockers and diuretics (standard therapy for heart failure) [2]. Nevertheless, the prognosis is poor, with reported mortality rates up to 15% and full recovery in only 23% of patients, while continuous deterioration is reported in up to 50% of patients despite optimal medical treatment [2-4]. Recently, oxidative stress-mediated generation of anti-angiogenic and pro-apoptotic 16 kDa prolactin and subsequent impaired cardiac microvascularisation have been related to peripartum cardiomyopathy [5]. A small pilot study suggested that prolactin blockade by bromocriptine in addition to standard therapy prevents repeated episodes of peripartum cardiomyopathy in patients presenting with a subsequent pregnancy [5]. Following this study, recent case reports have suggested that bromocriptine may also be beneficial in acute peripartum cardiomyopathy [6-8]. A survey of more than 1400 pregnant women who took bromocriptine primarily during the first few weeks of pregnancy found no evidence of increased rates of spontaneous abortion or congenital malformations [9]. However, safety issues were raised for patients taking bromocriptine in the early postpartum phase: a few case reports describe an increased risk of thrombotic events, such as myocardial infarction and retinal vein occlusion, in these patients [10-12].

## Case presentation

### Case report 1

**Figure 1:**
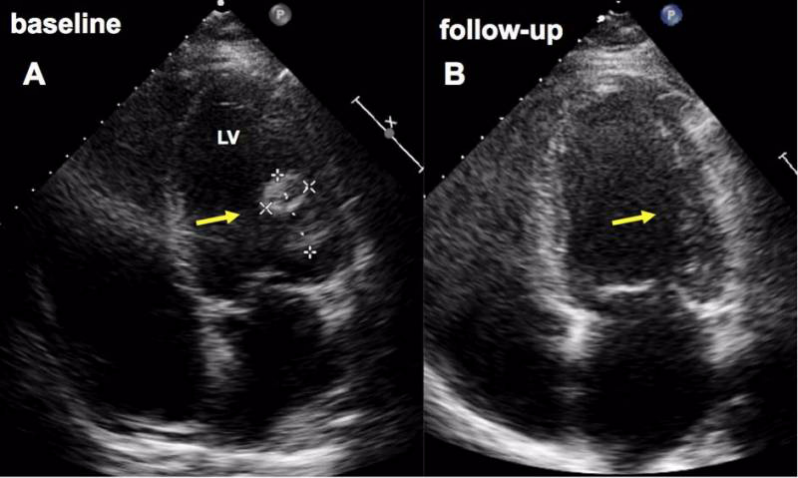
**Four-chamber view on initial echocardiogram demonstrates a large thrombus (arrow) attached to the lateral wall of the left ventricle (LV) at baseline (A), which has completely resolved after two months (B)**.

A 35-year-old African woman was admitted to our clinic four weeks after an elective caesarean section with chest pain, dyspnea, dry cough and New York Heart Association (NYHA) functional class IV. It was her third pregnancy and she had three healthy children. Her body mass index was 44. She had no pre-existing cardiac disease or exposure to cardiotoxic agents. Because of elevated D-dimer levels (15,44 μg/l) she was sent for computed tomography scans of her chest, which confirmed a suspected pulmonary embolism. The patient was therefore treated with the low-molecular-weight heparin (LMWH) enoxaparin in a therapeutic dose (0.1 ml/10 kg KG), before being switched to Coumadin (warfarin) (target international normalized ratio [INR] 2.5). Echocardiography revealed severe left ventricular dysfunction with an ejection fraction (left ventricular ejection fraction in percent; LV-EF) of 9%, left ventricular dilatation (left ventricular end diastolic diameter [LVEDD] 63 mm) and a thrombus of 37 × 23 mm (Figure 1). Peripartum cardiomyopathy was diagnosed, whereupon standard heart failure therapy was initiated; the patient was also treated with bromocriptine (5 mg/d for two weeks). Her blood work showed that 76 amino acid N-terminal fragment of Brain natriuretic peptide (NT-proBNP) (8084 ng/l) and C-reactive protein (CRP) (60 g/l) were markedly elevated, serum creatinine (102 umol/l) and Troponin T (TNT) (0.05 μg/l) were mildly elevated, and creatine kinase (CK) (59 U/l) was within normal range. Echocardiogram (ECG) showed sinus tachycardia (heart rate 115 beats/min) and no abnormalities indicative of repolarisation. Cardiac magnetic resonance imaging (MRI), taken 7 days after the ECG, confirmed left ventricular enlargement and severely impaired systolic left ventricular function (LV-EF 20%). It also revealed pericardial and pleural effusions. After three weeks, her left ventricular function and heart failure symptoms improved, accompanied by a decrease of NT-proBNP concentrations (1757 ng/l) and a decrease in size of the left ventricular thrombus (6 × 9 mm). During follow-up ECGs, the thrombus was no longer apparent (Figure 1). The patient was treated with bromocriptine (2.5 mg/d) for six weeks in total, while standard heart failure therapy plus Coumadin (warfarin) was continued for six months. After six months, left ventricular function had further improved (ECG indicated LV-EF 38%; MRI indicated LV-EF 45%), with regression of the pleural and pericardial effusions. Her NHYA class improved from IV to II, and her NT-proBNP serum level decreased to 261 ng/l.

### Case report 2

The sister of our original patient, aged 27, presented during her second pregnancy after developing peripartum cardiomyopathy. She had previously given birth to one healthy child via elective caesarean section, and had a body-mass index (BMI) of 30.4. Caesarean section was indicated due to imminent fetal asphyxia and amniotic infection syndrome. ECG revealed severe left ventricular dysfunction with an LV-EF of 32%, slight left ventricular dilatation (LVEDD 60 mm) and mild mitral regurgitation, but no pathological signs. NT-proBNP (7397 ng/l) and CRP (219 g/l) were markedly increased, serum creatinine (88 umol/l) and TNT (0,05 μg/l) were mildly elevated, and CK (29 U/l) was within normal range. Treatment with standard heart failure therapy, a half-therapeutic dose of enoxaparin (0.4 ml/d) for eight weeks and bromocriptine (5 mg/d for two weeks, then 2.5 mg/d for six weeks) was initiated. Eight months later, our patient had improved cardiac function (ECG LV-EF 47%; MRI LV-EF 59%), normal left ventricular dimensions and NT-proBNP (142 ng/l) within normal range.

## Discussion

Intraventricular thrombi diagnosed by ECG are not unusual in patients with peripartum cardiomyopathy [13]. It should be noted that there is generally an increased risk for thrombotic complications in peripartum women due to changes in coagulation activity in maternal blood characterized by elevation of factors VII, X and VIII, fibrinogen, and von Willebrand factor, which is maximal around term [14]. In peripartum cardiomyopathy patients with impaired cardiac function, the risk of thrombotic events is further increased. Our patient in case 1 presented with pulmonary embolism, acute heart failure and a large left ventricular thrombus at baseline. Upon initiation of standard therapy for heart failure together with heparin/Coumadin (warfarin) and bromocriptine, our patient steadily improved and the left ventricular-thrombus degraded without thrombotic complications. Our patient in case 2 was given low dose heparin during bromocriptine treatment and recovered fully without any adverse events. In fact, we have never observed thrombotic events in peripartum cardiomyopathy patients treated with bromocriptine and heparin [7] (this has also been confirmed by co-author Karen Sliwa in her unpublished observations of more than 30 peripartum cardiomyopathy patients). Bromocriptine treatment, in combination with heparin, appears not to increase the risk of thrombotic events in peripartum cardiomyopathy patients. Moreover, cardiac function and NYHA stage steadily improved in both of our patients, suggesting that bromocriptine with appropriate anti-thrombotic therapy is safe even in peripartum cardiomyopathy patients with manifested thrombosis at baseline.

While the risk of ventricular thrombi in peripartum cardiomyopathy seems more associated with the degree of cardiac dysfunction, the observation that bromocriptine was effective in both patients points to a similar pathophysiology of the disease in both sisters. Reports of peripartum cardiomyopathy with a positive family history are rare [1]. However, the higher incidence of peripartum cardiomyopathy in women with African ancestry suggests the presence of genetic risk factors. In this case report, we present peripartum cardiomyopathy in two sisters whose genetic history - a Caucasian mother with no history of peripartum cardiomyopathy and an African father - suggest a possible genetic predisposition to peripartum cardiomyopathy in women of African ancestry.

## Conclusion

Our case report suggests that bromocriptine with appropriate anti-thrombotic therapy is safe and beneficial even in peripartum cardiomyopathy patients with manifested thrombosis at baseline. Furthermore, the development of peripartum cardiomyopathy in two sisters suggests that women with a positive family history of peripartum cardiomyopathy might be at a higher risk for developing the disease. Pregnant women with peripartum cardiomyopathy should be monitored carefully for cardiac abnormalities during pregnancy and peripartum.

## Consent

Written informed consent was obtained from both patients for publication of this case report and accompanying images. A copy of the written consent is available for review by the Editor-in-Chief of this journal.

## Competing interests

The authors declare that they have no competing interests.

## Authors' contributions

GP performed MRI and data analysis, and contributed to the manuscript. SL performed data collection analysis. EP performed ECGs and monitored our patients. SK contributed to the manuscript. HD is chief of the Department of Cardiology and Angiology and supervised care of the patients and contributed to the manuscript. DH managed and organized data achievement and registry and contributed to the manuscript. All authors have read and approved the final manuscript.
